# Hypoplasia of cerebellar afferent networks in Down syndrome revealed by DTI-driven tensor based morphometry

**DOI:** 10.1038/s41598-020-61799-1

**Published:** 2020-03-25

**Authors:** Nancy Raitano Lee, Amritha Nayak, M. Okan Irfanoglu, Neda Sadeghi, Catherine J. Stoodley, Elizabeth Adeyemi, Liv S. Clasen, Carlo Pierpaoli

**Affiliations:** 1 Drexel University, Department of Psychology, Philadelphia, PA 19104 USA; 2 National Institute of Biomedical Imaging and Bioengineering, NIH, Quantitative Medical Imaging Section, Bethesda, MD 20892 USA; 3 American University, Department of Psychology, Washington, DC 20016 USA; 4 Alabama College of Osteopathic Medicine, Dothan, AL 36303 USA; 5 National Institute of Mental Health, NIH, Developmental Neurogenomics Unit, Human Genetics Branch, Bethesda, MD 20892 USA

**Keywords:** Diffusion tensor imaging, Developmental disorders

## Abstract

Quantitative magnetic resonance imaging (MRI) investigations of brain anatomy in children and young adults with Down syndrome (DS) are limited, with no diffusion tensor imaging (DTI) studies covering that age range. We used DTI-driven tensor based morphometry (DTBM), a novel technique that extracts morphometric information from diffusion data, to investigate brain anatomy in 15 participants with DS and 15 age- and sex-matched typically developing (TD) controls, ages 6–24 years (mean age ~17 years). DTBM revealed marked hypoplasia of cerebellar afferent systems in DS, including fronto-pontine (middle cerebellar peduncle) and olivo-cerebellar (inferior cerebellar peduncle) connections. Prominent gray matter hypoplasia was observed in medial frontal regions, the inferior olives, and the cerebellum. Very few abnormalities were detected by classical diffusion MRI metrics, such as fractional anisotropy and mean diffusivity. Our results highlight the potential importance of cerebro-cerebellar networks in the clinical manifestations of DS and suggest a role for DTBM in the investigation of other brain disorders involving white matter hypoplasia or atrophy.

## Introduction

Down syndrome (DS), the most common genetic cause of intellectual disability^1^, is characterized by prominent impairments in language^2^, memory^3^, motor^4^, and executive function^5^. However, the neural correlates of these impairments are poorly understood, particularly in pediatric samples. Surprisingly little is known about the developing DS neuroanatomic phenotype studied *in vivo*, as most research has targeted older participants given high rates of precocious-onset Alzheimer’s disease (AD) in this group^6^.

A review of the DS pediatric structural neuroimaging literature reveals a small corpus of studies that largely report on gross anatomical deviations in targeted brain structures or the whole brain using manual or semi-automated segmentation on conventional (T1) magnetic resonance imaging (MRI) scans. More recently, researchers have adopted finer-grained approaches to describe neuroanatomy in pediatric DS samples^7–9^. However, there are only four published DTI studies – one focused on toddlers^10^ and three focused on adults^11–13^. Detailed DTI investigations during childhood, adolescence, and young adulthood are desirable, given the importance of this developmental period within the context of typical brain and cognitive development^14^. This is particularly relevant for DS, as the disorder is characterized by slowed cognitive development during childhood that tapers in young adulthood^15^ and precedes the cognitive decline associated with precocious-onset AD in a subset of individuals with the syndrome^16^. Thus, augmenting our understanding of the DS neuroanatomic phenotype during this period may help generate hypotheses about the neural underpinnings of this cognitive slowing and risk for AD and provide insights into interventions that could alter this trajectory.

Previous pediatric imaging studies have described several global neuroanatomic alterations in DS, including reduced total brain^8,9,17–19^ and gray and white matter volumes^8,9,17,19^ as well as more specific volume, density, and surface area reductions in the frontal and temporal lobes^7–9,18^, hippocampus^19,20^, and cerebellum^7,10,17,21,22^. However, there are no detailed investigations of white matter in this group, resulting in little understanding of specific white matter pathways that are most impacted in DS. Typical whole-brain MRI morphometry studies use T1-weighted images (T1WIs) to measure voxelwise differences in volume using a technique known as tensor based morphometry (T1-TBM)^23,24^. However, T1-TBM may not be adequate for assessing the volume of specific pathways in white matter regions that appear homogeneous on T1WIs. In these regions, white matter pathways are identifiable by DTI^25^.

Thus, in the current study we utilized DTI to provide a detailed, whole-brain description of the DS neuroanatomic phenotype during childhood and young adulthood. We report both tract-based spatial statistics (TBSS) as well as a whole-brain analysis (using threshold free cluster enhancement [TFCE] analyses) of classical DTI metrics including fractional anisotropy (FA)^26^ and mean diffusivity (MD)^27^ that have been shown to be sensitive to microstructural and organizational features of brain tissue. In addition, to characterize morphometric abnormalities, we utilized a recently-developed approach which performs TBM using deformation fields that are constructed using all scalar and directional information contained in the diffusion tensor. This technique, called DTI-driven tensor based morphometry (DTBM^28^), is particularly valuable for detecting morphological abnormalities of specific white matter pathways and complements traditional FA and MD measures.

## Methods

All data were collected at the National Institutes of Health (NIH) Clinical Center in Bethesda, MD. Procedures were approved by the NIH Combined NeuroScience Institutional Review Board and executed in accordance with their guidelines and those of the NIH. Informed consent was obtained consistent with guidelines provided in the Declaration of Helsinki and NIH policies. See Lee *et al*.^8^ for details.

### Participants

The participants included in the present study’s analyses were recruited as part of a DS pediatric neuroimaging research program^8^. All participants with DS were diagnosed with Trisomy 21 (see Lee *et al*.^8^ for details). To be included in the present study’s analyses, participants also needed to have completed DTI and T1-weighted image acquisition with acceptable image quality. Fifteen participants with DS were included in the present study.

In addition, 29 typically developing (TD) individuals were included. Fifteen of these 29 TD participants served as individually age- and sex-matched controls for the 15 participants with DS. The scans of the remaining 14 were used to create independent control population templates for image analysis. TD participants were recruited using procedures outlined in Giedd *et al*.^14^; they were screened by phone prior to study enrollment to exclude psychiatric/learning difficulties and acquired brain injury.

The DS (n = 15) and TD (n = 15) groups were matched on age, (p > 0.7), sex (p > 0.9), and socioeconomic status^29^ (p > 0.8). However, they differed, as expected, on IQ (TD > DS), t(28) = 9.02, p < 0.001. See Table 1. For details about the participants included in the larger research program, see Supplemental Material.

**Table 1:** Demographic characteristics of the sample.

	DS (n = 15)			TD Controls (n = 15)		
	M	SD	Range	M	SD	Range
Age	17.0	5.5	6–23	17. 8	6.1	6–24
Nonverbal IQ	59.5	15.2	34–86	115.8	18.8	81–151
SES (Hollingshead)^a^	34.5	15.1	20–63	33.4	13.4	20–51
	**N**	**%**		**N**	**%**	
Sex: Female	7	46.7		7	46.7	

^a^Note: SES data are missing for 2 participants in the control group. The racial/ethnic breakdown of the groups was as follows: Asian (DS: n = 1; TD: n = 1), Black (DS: n = 2; TD: n = 1), Hispanic (DS: n = 1; TD: n = 1), White (DS: n = 8; TD: n = 12), Other (DS: n = 3; TD: n = 0).

### Image acquisition and processing

All scans were completed without sedation on a 3-Tesla General Electric Scanner with an 8-channel head coil. DTI scans consisted of 60 diffusion weighted volumes with 6 b = 0, 12 b = 300, and 42 b = 1100 s/mm^2^. The resolution was 2.5 mm isotropic zero-filled at the scanner to 1.87 × 1.87 × 2.5 mm.

Diffusion weighted images (DWIs) were assessed for artifacts using the quality assessment criteria applied in the NIH MRI study of normal brain development DTI database^30^. T2WIs were anterior commissure-posterior commissure (AC-PC) aligned^31^. Datasets were processed to correct for eddy, motion, and EPI distortion effects using version 2.5.2 of the TORTOISE DTI processing software^32^. For each participant, from the corrected DWIs, the diffusion tensor (DT) was computed using weighted nonlinear fitting^33^. From the diffusion tensor, FA, MD, and directionally encoded color (DEC) maps^25^ were computed using TORTOISE^32,34^.

T1-Weighted Images (T1WIs) were collected with a 3D gradient echo sequence (resolution 0.94 × 0.94 × 1.2 mm) with the following parameters: 128 slices; 224 × 224 acquisition matrix; flip angle = 12°; field of view [FOV] = 240 mm. T1WIs were rigidly registered^35^ to AC-PC aligned T2WIs.

#### Template creation

As stated above, within our TD control population (n = 29), 15 individuals were matched to the DS group and served as controls. Average brain templates for both T1WIs and DT were created from the remaining 14 controls. These templates served as a target for spatial normalization of individual participant data for both the DS and TD groups (n = 15 each). The DT template was computed using the DR-TAMAS registration software^36^. (See Supplemental Table 1 for template group demographic characteristics and Supplemental Fig. 1 for magnitude difference maps for each brain metric between the TD control and study template groups. These difference maps were generated to verify that there were no large differences between these two samples of the TD population.)

DT computed for DS and TD participants from their native space was then registered to the DT template using DR-TAMAS. Spatially normalized FA, MD, and DEC maps were derived from the spatially normalized tensors. Spatially normalizing tensors aims to remove individual morphological differences (making them suitable for voxelwise analysis of DTI derived quantities) and allows evaluation of the deformation fields to perform tensor-based morphometry.

For DTI-driven tensor based morphometry (DTBM) analyses, the Logarithm of the determinant of the Jacobian (Ln-J) of the deformation applied to bring each individual tensor into the DT template space was computed in a voxelwise manner. Ln-J provides information about the size of a particular structure in each participant (either DS or TD) relative to the average size of that structure in the population that has been used to build the group template (in this case, the independent set of 14 controls). A negative Ln-J value signifies that the structure in the participant is smaller than the average volume for that structure in the study template control group (hypoplasia) while a positive value indicates the opposite. An analogous procedure was used for the tensor based morphometry analysis of T1-weighted images, with the exception that the ANTS software^35^ was used for template creation and spatial normalization.

#### Region of interest analyses

To quantify morphometric differences for gray matter ROIs, Ln-J mean values for the DS and TD groups were computed in the space of the DT template for 43 ROIs. The regions considered are Desikan atlas^37^ regions included in the IIT v4.2 T1W template^38,39^. An ROI for the inferior olive, a structure not defined in the Desikan atlas, was added by the authors. Registration of the IIT v4.2 ROIs to the DT template was accomplished as follows: The T1W template, which is virtually coregistered to the DT template, was registered to the IIT 4.2 T1W template, using ANTS SyN registration^35,40^, and the transformation resulting from this registration step was then applied to each individual Ln-J map, permitting assessment of ROI-specific Ln-J values. However, the large cortical ROIs of the Desikan atlas include voxels that are highly contaminated by CSF. To report Ln-J values that pertain to voxels containing brain tissue and not CSF, we excluded voxels with high CSF content. Specifically, we performed in each participant’s native space a dual tensor fitting as previously described by Pierpaoli *et al*.^41^. This procedure produces a “CSF signal fraction” measure in each voxel, that can be used to eliminate from the analysis voxels that are largely contaminated by CSF. Only voxels with a CSF signal fraction lower than 30% were included in the morphometric analysis.

White matter ROIs were defined directly on the DEC maps of the DT template (Supplemental Fig. 2). They included most of the regions described in the JHU white matter atlas^42,43^ plus additional regions relevant for this study but not defined on the JHU atlas (e.g., anterior and posterior transverse pontine fibers; lateral, intermediate, and medial aspects of cerebral peduncles). We did not attempt to warp the JHU atlas ROIs directly because spatial co-registration of JHU ROIs with a population-specific template was found to be suboptimal in previous studies^44^. Since CSF contamination is less likely for narrowly defined white matter ROIs, we did not perform the CSF elimination step that was used for the gray matter ROIs.

For the purposes of presenting these data, Ln-J values were calculated for both the DS and TD groups relative to the template. Then the difference between these values was calculated (DS-TD mean) and presented in bar graphs described further in the Results section. Note: average Ln-J values for the left and right hemispheres are reported.

#### Statistical procedures

Statistical analysis of group differences for FA, MD, T1-TBM, and DTBM was performed using both the tract-based spatial statistics (TBSS) and threshold free cluster enhancement (TFCE) tools provided in the FSL randomize toolkit^45,46^. TBSS analysis, which requires skeletonization of white matter, in our view, is unnecessary given the excellent spatial normalization obtained with DR-TAMAS; however, we included this analysis for comparison with previous literature. Statistics were performed with 10,000 permutations. Results are presented on statistical significance maps generated with a threshold of p < 0.01; these results were corrected for multiple comparisons using a family‐wise error rate of 1% (p  <  0.01). In addition to statistical significance maps, magnitude difference maps (DS mean – TD mean) for all metrics were generated. Lastly, group differences between patients and controls in mean within ROI Ln-J values were assessed in several gray and white matter ROIs using t-tests with Bonferroni correction for multiple comparisons.

#### Tractography procedures

In order to investigate morphometric features of specific white matter pathways, the AFNI’s, opensource, FATCAT software^47–49^ was utilized to compute tracts from seed regions that showed an interesting volumetric pattern in the Ln-J magnitude difference maps and in the statistical analysis maps.

## Results

**Figure 1:**
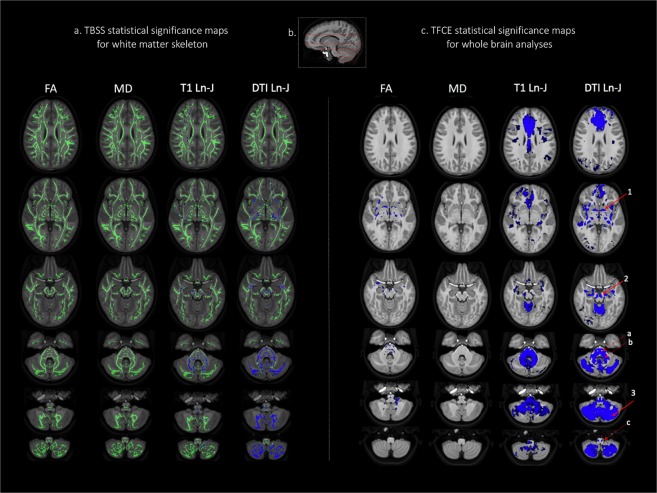
Statistical significance maps from tract-based spatial statistics (TBSS) and threshold free cluster enhancement (TFCE) analyses for FA, MD, T1-TBM and DTBM for the DS-TD group comparisons. Panel a includes TBSS results in axial view; Insert b provides a reference for axial slices that appear in this figure (as well as Fig. 2). Panel c displays TFCE results. Blue color denotes DS < TD (p < 0.01). Note, in Panel c, solid arrows are included to highlight some of the structures that were identified to be statistically hypoplastic using DTBM but not T1-TBM. These examples include (1) the anterior commissure, (2) the medial portion of cerebral peduncle, and (3) a large swath of the cerebellum. We also highlight three instances in which DTBM analyses more clearly differentiated between hypoplastic and normal structures using dotted arrows. Specifically, we draw the reader’s attention to the fact that the (a) corticospinal tract is not significantly hypoplastic in DTBM, but as can be seen, the (b) transverse pontine fibers are clearly hypoplastic. In addition, we highlight selective hypoplasia of (c) the inferior olives in the medulla that is detected by DTBM and not T1-TBM.

Figure 1 (Panel a) and Table 2 report the TBSS analysis results for differences between the DS and TD groups on the white matter skeleton. For FA and MD (Fig. 1, Panel a, columns 1, 2), there were no group differences. TBM analysis (Fig. 1, Panel a, columns 3, 4) showed significant group differences (DS < TD) along the white matter skeleton for T1 and diffusion data, particularly in the cerebellum. However, the number of white matter skeleton voxels that differed significantly was much higher for DTBM than T1-TBM (21% vs. 9%, respectively).

**Table 2:** Percentage of significantly different voxels by approach (p < 0.01).

	TBSS-skeleton^a^				TFCE-voxelwise^b^			
	FA	MD	T1-TBM Ln-J	DTBM Ln-J	FA	MD	T1-TBM Ln-J	DTBM Ln-J
DS < TD	0.0	0.0	9.9	21.4	1.0	0.0	16.9	18.5
DS > TD	0.0	0.0	0.0	0.0	0.0	0.0	0.0	0.0

^a^Total skeleton voxels: 118,311; ^b^Total brain voxels: 1,248,880.

Whole brain, voxelwise (TFCE) analyses were also completed. For these analyses, brain maps display the statistical significance of these differences in Fig. 1 (Panel c). Similar to the TBSS analyses, these analyses revealed very few group differences in FA and MD. Significant differences for FA were found in 1% of brain voxels and no difference was found for MD analyses (Table 2). Small clusters of reduced FA were observed in the inferior and middle cerebellar peduncles, cerebral peduncles, external capsule, anterior corona radiata, internal capsule, fornix, and medial lemniscus.

In contrast, TBM analyses revealed regional hypoplasia for the DS group throughout the brain. The percentage of voxels with statistically significant hypoplasia detected by T1-TBM and DTBM was relatively similar (16.9% and 18.5%, respectively). However, DTBM appeared to have higher anatomical specificity than T1-TBM, being able to localize the volume abnormality of specific pathways in a given white matter region. For example, the pons in DS was homogeneously hypoplastic in T1-TBM analysis. In contrast, DTBM revealed that the descending motor pathways had normal volume, while the transverse pontine fibers were severely hypoplastic. This higher anatomical specificity of DTBM versus T1-TBM can be observed in other regions throughout the brain, including the medulla where DTBM revealed selective hypoplasia of the olives in DS. In addition, other regions of hypoplasia detected by DTBM and not detected by T1-TBM include the anterior commissure, the medial aspect of the cerebral peduncle, and a large swath of the cerebellum.

**Figure 2:**
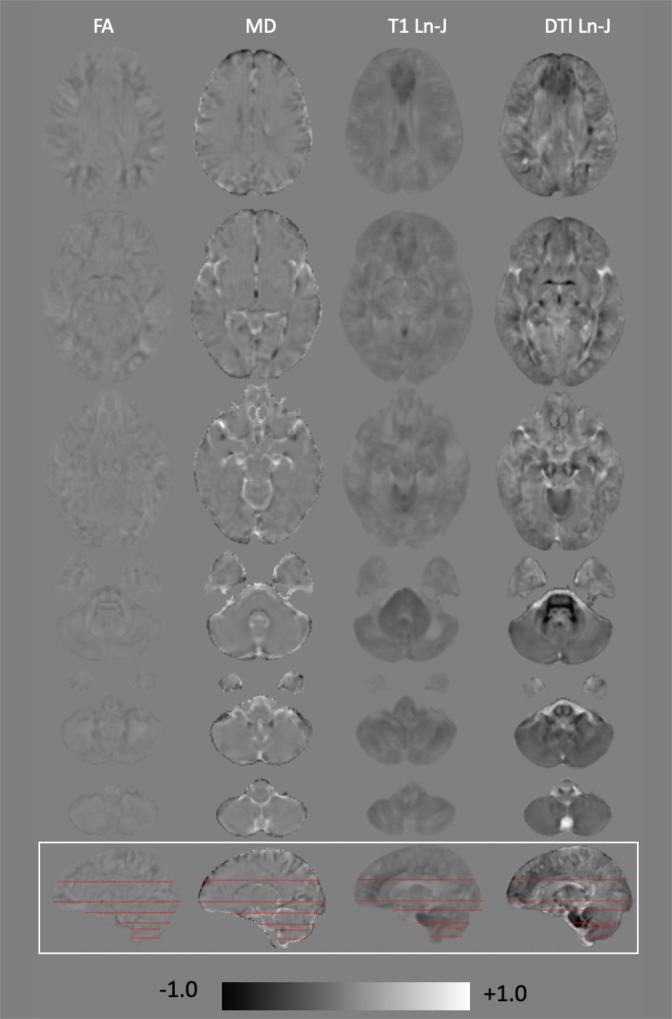
Magnitude difference for DS-TD group for FA, MD, T1-TBM and DTBM. The gray level in the background correspond to zero. Gray levels darker than the background indicate negative values (DS < TD) and gray levels brighter than background indicate positive values (DS > TD). All values are scaled from -1 (black) to +1 (white). Ln-J and FA values are dimensionless, MD values are in units of 10^-3^ mm^2^/s.

In Fig. 2, whole brain, magnitude difference maps are presented – the first two columns display the magnitude difference maps for FA and MD; the second two columns display Ln-J magnitude differences maps for T1 and diffusion images. The gray background corresponds to 0 – i.e., no differences between the DS and TD groups. Darker gray shades denote negative values for the comparison (i.e., lower FA and MD; hypoplasia in DS); brighter gray shades indicate positive values (i.e., higher FA and MD; larger volume in DS). The greater anatomical specificity of DTBM over T1-TBM can be seen by contrasting the magnitude differences maps in columns 3 (T1) and 4 (DTI) of this figure.

Given the higher anatomical specificity demonstrated by DTBM, in the reminder of the manuscript we will focus on the results of the DTBM analysis, rather than on those of the T1-TBM analysis. DTBM revealed significant hypoplasia of the cerebellar hemispheres, pons (including transverse pontine fibers), inferior olivary nuclei, inferior and middle cerebellar peduncles, and a large region of the medial superior frontal cortex. Within the cerebellum, the anterior lobe and medial posterior regions (lobules IX, X) showed marked hypoplasia, even relative to the other regions of the cerebellar cortex. Lastly, significant hypoplasia was observed in the fornix and the cingulate.

### ROI Results

**Figure 3:**
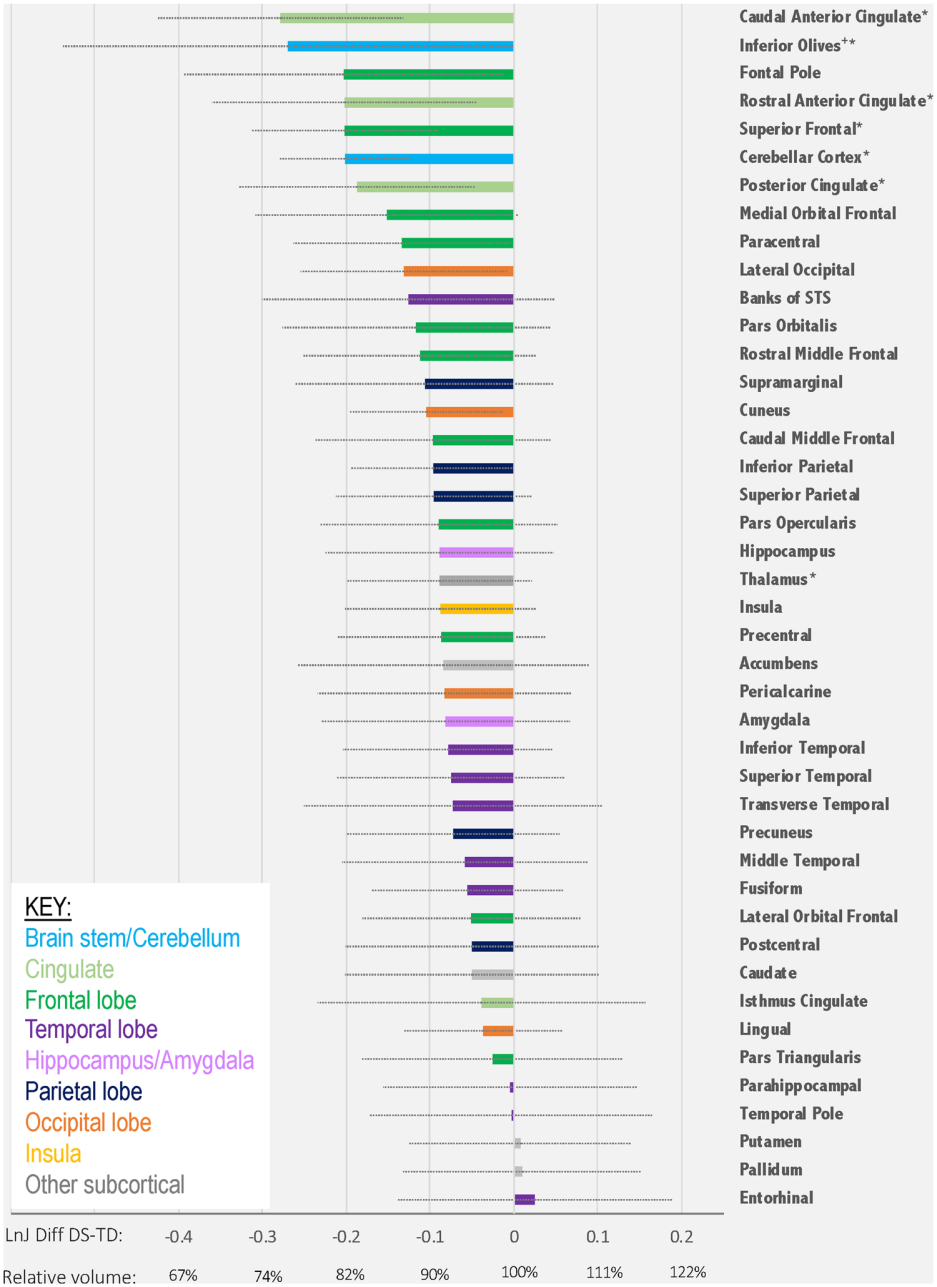
Volume of gray matter regions in the DS group relative to the volume of the same regions in the TD group. Following registration into the DT template, Ln-J mean values for the DS and TD groups were computed in 42 gray matter structures reported in the IIT 4.2 T1W atlas and in the inferior olives (noted with a ^+^ sign). For each region, we computed the mean Ln-J difference between DS and TD. These difference values were ranked from most negative (DS smaller volume than TD) to positive (DS larger volume than TD) and are color-coded by region. Error bars represent the standard deviation of the Ln-J for the DS group. On the X-axis, we report the Ln-J difference as well as the average volume for the DS group expressed as percentage of the average volume of the TD group. In addition, we note with an asterisk regions in which t-tests comparing that ROI’s volume between groups were statistically significant (p < 0.05) following Bonferroni correction for multiple comparisons. Note: Banks of STS = Banks of the Superior Temporal Sulcus.

For each gray matter ROI (described in the Methods), the mean Ln-J for the TD group (relative to the template) was subtracted from the DS mean (relative to the template). These values were then ranked from most negative (most hypoplastic) to positive (hyperplastic). These results are presented in Fig. 3. T-tests with Bonferroni correction for multiple comparisons detected several subregions of the cingulate cortex, the cerebellum, the superior frontal gyrus, the inferior olives, and the thalamus as significantly smaller in the DS than TD group.

**Figure 4:**
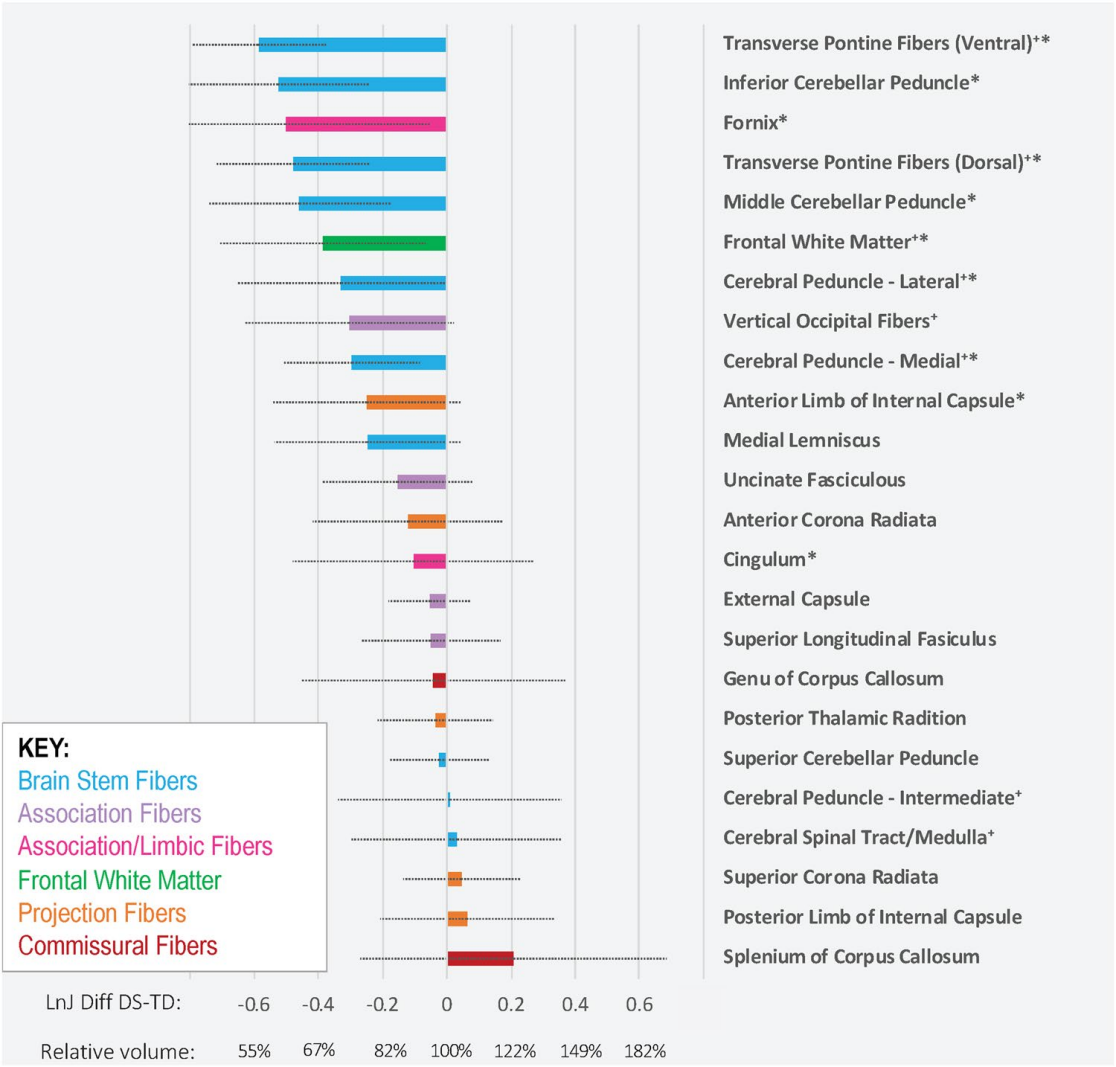
Volume of white matter regions in the DS group relative to the volume of the same regions in the TD group. Following registration into the DT template, Ln-J mean values for the DS and TD groups were computed for most of the regions described in the JHU white matter atlas (Oishi *et al*. 2008) plus additional regions (noted with a ^+^ sign) relevant for the current investigation. Then the difference in Ln-J for the two groups was calculated (DS Ln-J mean – TD Ln-J mean). These difference values were ranked from most negative (DS smaller volume than TD) to positive (DS larger volume than TD) and are color-coded by the pathway type. Error bars represent the standard deviation of the Ln-J for the DS group. On the X-axis, we report the Ln-J difference as well as the average volume for the DS group expressed as percentage of the average volume of the TD group. In addition, we note with an asterisk regions in which t-tests comparing that ROI’s volume between groups were statistically significant (p < 0.05) following Bonferroni correction for multiple comparisons.

The magnitude of the difference between the DS and TD groups was also calculated for white matter ROIs. These values are presented in Fig. 4 and are ranked from most hypoplastic to hyperplastic. Bonferroni-corrected t-tests revealed group differences in the transverse pontine fibers, inferior and middle cerebellar peduncles, medial and lateral aspects of the cerebral peduncles, frontal white matter, the fornix, and the cingulum (DS < TD).

### Tractography results

**Figure 5:**
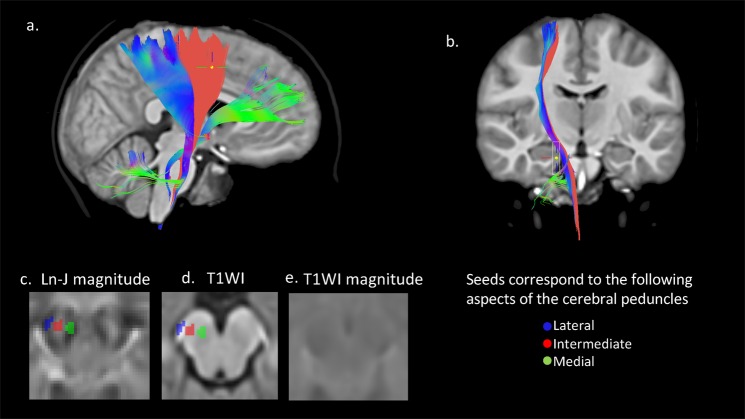
Tractography of different subregions of the cerebral peduncle. Panels a and b show the deterministic tractography results computed using the ROIs of the cerebral peduncle subdomains. Three ROIs are defined manually on the cerebral peduncle using the DTI Ln-J magnitude difference map as a reference (panel c). Also shown is the anatomical reference of the ROIs on the T1WI (*panel d*) as well as the magnitude difference map for the T1W1 (panel e). As can be seen in panel c, the lateral (blue seed) and medial (green seed) aspects of the cerebral peduncle correspond to regions of hypoplasia in the DS population. The intermediate aspect (red seed) corresponds to a region of normal volume in the DS population (as evidenced by its lighter shade). This intermediate region gives origin to fibers projecting to the motor cortex (anterior to the central sulcus). The medial region (green) gives origin to fibers projecting to the ventro-medial frontal lobe (Arnold’s bundle), while the seed in the lateral aspect (blue) gives origin to fibers projecting to the parietal lobe (Turck’s bundle).

As seen in Fig. 4, a peculiar volumetric pattern was observed in the cerebral peduncles of participants with DS: significant hypoplasia in the medial and lateral aspects and normal volume in the intermediate aspect. Figure 5 illustrates deterministic tractography results for seeds in these three aspects of the cerebral peduncles – the lateral (blue), medial (green), and intermediate (red) aspects. Tractography results indicate that the intermediate, morphometrically normal region, contains fibers projecting to the motor cortex (anterior to the central sulcus), while the medial and lateral regions that showed severe hypoplasia contain fibers projecting to the ventro-medial frontal and temporo-parietal-occipital cortices, respectively.

**Figure 6:**
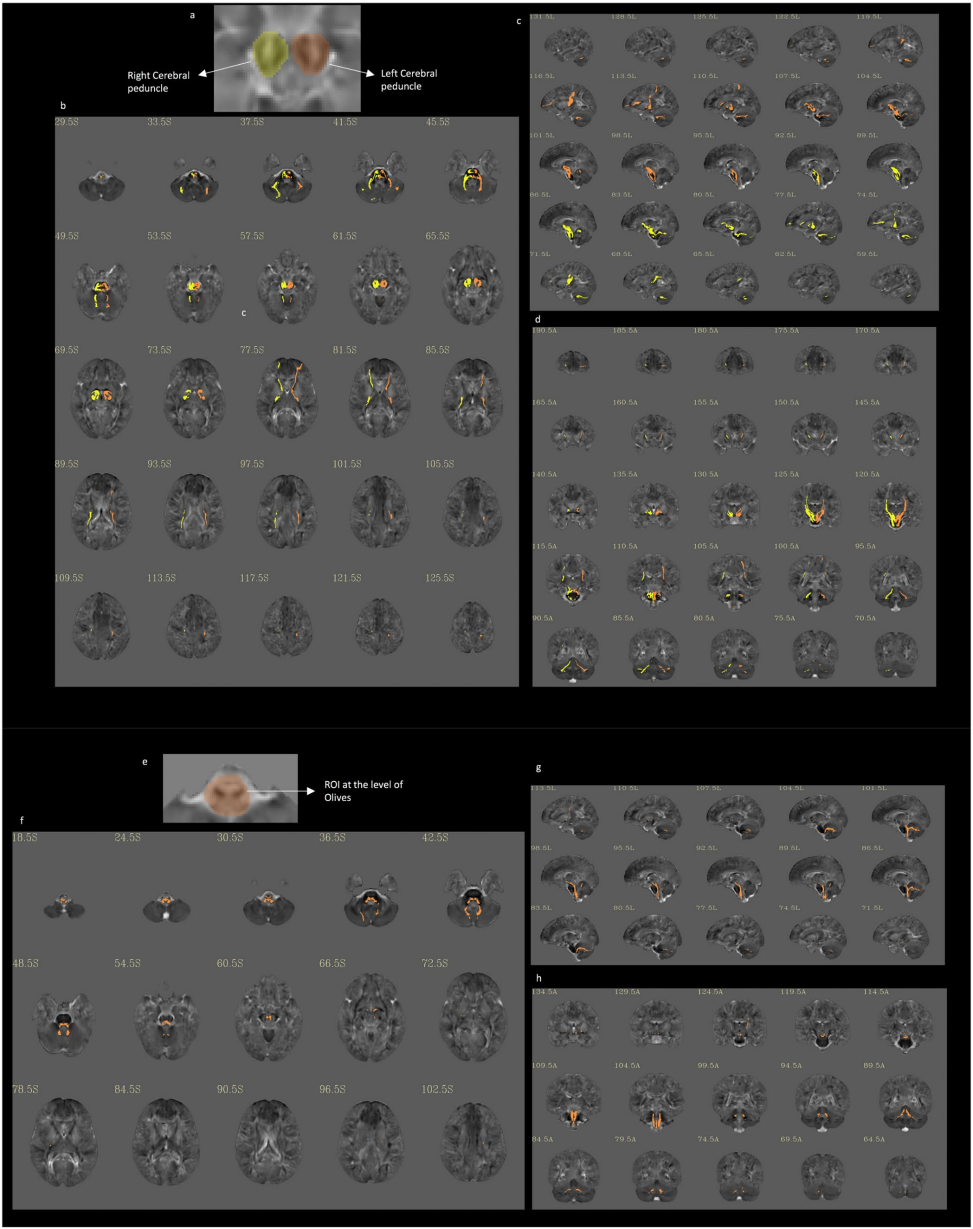
Extraction of pathways hypoplastic along their entire trajectory. Right (yellow) and left (orange) ROIs encompassing the entire section of the cerebral peduncle are drawn as shown in Panel a. Deterministic tractography is performed from the seeds, but only trajectories that run in voxels with Ln-J magnitude values <0 (hypoplasia in the DS group) are shown in Panels b–d. This approach allows the extraction of pathways that pass through the seed regions and are consistently hypoplastic along their entire trajectory. Panels f–h show similarly extracted tracts from a large seed region at the level of the inferior olives (ROI shown in Panel e).

Figure 6 displays tractography results obtained for tracts that run for their entire trajectory through voxels having negative Ln-J values – i.e., *voxels that were hypoplastic in DS for their entire trajectory*. This was done to extract and document the anatomical continuity of hypoplastic pathways in DS subjects. For example, when we seeded the entire cerebral peduncles (i.e., medial, intermediate, and lateral aspects) but only pathways running through voxels that had a negative Ln-J value (i.e., DS < TD voxels) to be displayed, we document (in panels b–d) that this feature extraction approach clearly highlights fronto-pontine-cerebellar pathways. Specifically, at levels 77, 81, and 85, hypoplastic frontal pathways can be seen; at levels 41, 45, 49, and 53, hypoplastic pathways running through the pons are evident; lastly, at 33, 37, and 41, hypoplastic tracts running through the cerebellum are evident. Also seen are hypoplastic temporo-occipito-parietal-pontine-cerebellar pathways in slices 110, 113, and 116.

Lastly, the right side of Fig. 6 (panels e–h) visualizes the hypoplastic olivocerebellar tract. In this case, the inferior olives were seeded and then the projected tractography results were displayed for tracts consistently running through hypoplastic voxels. Levels 36, 42, 101, and 104 illustrate the hypoplasia of fibers connecting the olives to the contralateral cerebellar cortex through the inferior cerebellar peduncles.

## Discussion

To date, only four DTI studies of DS have been published– one of toddlers and three of adults/older adults^10–13^. Our study is the first to investigate school-age children and young adults with DS. Thus, this study fills an important developmental gap in the literature by investigating brain features during a time in which dynamic changes in the brain anatomy and behavior are known to occur^14^ and before the onset of age-related neurodegeneration. In addition, our study is novel in that it not only analyzed classical DTI metrics, such as FA and MD, but we also utilized DTI to perform a morphometric study, characterizing regional and pathway-specific volumetric differences between participants with DS and TD controls.

Findings from this investigation have advanced our understanding of the DS neuroanatomic phenotype during this important developmental period in several ways. First, very few differences in FA and MD were observed between the DS and TD groups. Indeed, the widely-utilized TBSS approach, which uses a “skeletonized” white matter representation, found no statistically significant differences in FA or MD. The whole-brain, voxelwise TFCE analysis revealed few regions with statistically significant FA differences and no regions with MD differences. FA and MD are sensitive indicators of compositional, microstructural, and local architectural abnormalities of brain tissue. Subtle changes in brain tissue features such as the degree of local fiber coherence, cellularity, edema, gliosis, Wallerian degeneration, and to a lesser extent myelination, are known to affect FA and MD profoundly^50^. Dramatic changes in FA and MD are also known to occur in typical brain development from birth to adulthood^30,51^. The almost complete normality of FA and MD values found here suggests that white matter structural tissue abnormalities as characterized by FA and MD are not a relevant feature of the DS neuroanatomical phenotype during the school-age and young adult period.

Our FA and MD results differ from those obtained by both Gunbey *et al*.^10^ and Romano *et al*.^11^ who studied toddlers and adults with DS, respectively. Both of these studies applied a more lenient statistical threshold than applied in the current paper. However, we were not able to replicate many of their findings even if we relaxed the threshold for statistical significance to p < 0.05. The inferior longitudinal fasciculus, inferior fronto-occipital fasciculus, and the anterior limb of the internal capsule that had significantly reduced FA in at least one of the earlier studies^10,11^ showed a similar trend in our study without reaching statistical significance (p ~0.15). All considered, we do not rule out white matter microstructural abnormalities in DS during childhood, adolescence, and young adulthood, particularly in frontal-subcortical circuits; however, our study’s findings suggest that these abnormalities are overall modest in magnitude and spatial extent, and less prominent than volumetric differences.

Morphometric analyses indicated very significant hypoplasia of both white and gray matter in multiple regions. Reduced volume in white matter may originate from a lower number of fibers or from fibers that have smaller diameter. Smaller fiber diameter could be consequent to hypomyelination, and hypomyelination only marginally affects anisotropy in the absence of edema or structural damage to the axon. While DTI-based morphometry cannot differentiate these underlying mechanisms, recent research reports hypomyelination of the neocortex in the Ts65dn mouse model of Trisomy 21^52^ as well as dysregulation of genes associated with oligodendrocyte development and myelination in brain tissue of humans with DS and the Ts65Dn mouse model. Therefore, transcriptome analyses of the brain tissue of humans with DS and murine models implicate proteins relevant to white matter development^52^. Although consistent with our volumetric findings, further studies are needed to assess if these mechanisms are the underpinning of our findings in a clinical population.

The current study’s T1-TBM findings confirm and extend prior investigations documenting widespread reduction of white matter volume in humans with DS using T1-W imaging^7,9^. Both T1-TBM and DTBM revealed a similar percentage of brain parenchyma that was significantly hypoplastic in DS; however, DTBM was much more specific than T1-TBM in identifying selective hypoplasia of specific white matter pathways in areas that would not stand out from the surrounding regions in T1-TBM maps. For example, while the cerebral peduncle appeared homogeneous in T1-TBM maps, DTBM revealed three distinct subregions with different degrees of hypoplasia. A lateral and medial aspect that were severely hypoplastic, and an intermediate aspect that was similar in volume to TD controls. The medial, lateral, and intermediate portions of the cerebral peduncle include fibers from different cortical regions^53^. Arnold’s and Turck’s bundles have been described to occupy the medial and lateral aspects of the cerebral peduncle, respectively^53,54^. Arnold’s bundle is believed to run from the frontal cortex to the pons^53,54^ from which originate fibers that project to the cerebellum through the middle cerebellar peduncle^53^. Turck’s bundle is believed to encompass retrorolandic projections, including those from the temporal, parietal, and occipital lobes^55^. Lastly, the intermediate peduncular region includes the cortico-spinal and cortico-bulbar tracts^55^. Remarkable hypoplasia in the medial (Arnold’s bundle) and lateral (Turk’s bundle) aspects of the cerebral peduncle was observed, while the intermediate aspect, which houses motor fibers, was similar in volume to TD controls. Consistent with the preservation of their volume at the level of the cerebral peduncle, the descending motor fibers had normal volume at all brainstem levels, including the pons and medulla. These findings indicate that the cortico-spinal pathway is relatively unimpaired in DS, indicating that other pathways, such as hypoplastic anterior regions of the cerebellum which support motor control, could be responsible for the significant motor impairments in DS^55^.

Another notable morphological finding involved the severe hypoplasia observed in pontine-cerebellar and olivocerebellar pathways in DS. These two afferent pathways to the cerebellum (via the middle and inferior cerebellar peduncles, respectively) along with their associated gray matter structures (including the medial prefrontal and cingulate cortex as well as the cerebellum, Arnold’s bundle, pons, and inferior olivary nuclei) were massively reduced in volume in DS. Although previous investigations have reported hypoplasia in the frontal lobes^7,19^, pons^56,57^, and cerebellum^7,10,17,21,22^, no studies have documented the degree to which these gray matter structures and their anatomical connections are impacted. Moreover, this is the first investigation to document severe hypoplasia of the inferior olivary nuclei in DS. The current study also provides a greater understanding of particular frontal and cerebellar subregions that are most atypical in DS. Within the frontal lobes, medial prefrontal regions along with more anterior regions of the cingulate gyrus showed the greatest hypoplasia. Within the cerebellum, significant hypoplasia of multiple regions of the cerebellar cortex thought to be involved with motor and cognitive functions^58–60^ was observed, consistent with the DS cognitive-behavioral phenotype in which prominent motor and cognitive deficits have been reported^55^.

In contrast to the hypoplasia observed in the inferior and middle cerebellar peduncles and the cerebellar cortex, less significant volume reductions were noted in the superior cerebellar peduncle. This dissociation in afferent (inferior and middle cerebellar peduncles, inferior olivary nuclei) vs. efferent (superior cerebellar peduncle) pathways suggests relative differences in the impact of DS on the pathways to and from the cerebellum. It has been proposed that cerebro-cerebellar circuits may critically shape brain development (see Stoodley^61^ for review), and so the significant hypoplasia observed in cerebro-cerebellar circuits in DS warrants further investigation.

While the neuroimaging literature on young people with DS is growing, we still know relatively little about brain development in DS and importantly which structures are atypical well before the onset of age-related neurodegeneration. In addition to the cerebro-cerebellar circuits described above, a novel finding from this investigation was the identification of significant hypoplasia of the fornix in the DS group. The fornix, a major white matter tract that relays information to and from the hippocampus, has not received much attention in the DS research literature. However, our finding that the volume of fornix was ~60% of that observed in TD participants suggests that the fornix should be investigated further in DS, given the prominent memory impairments that characterize the syndrome (for review, see Jarrold *et al*.^62^). The fornix may also be relevant to aging in DS, as it has been implicated in the early identification of AD in the general population^63^.

The current study has some limitations, one being its small sample size, which precluded the evaluation of the age-related trajectory of brain morphometry deviations in DS. This is an important step for future research, given the slowing of cognitive development that occurs in DS during childhood and adolescence^15^ and the precocious onset of AD that occurs for many with the syndrome^16^. Studies of neuroanatomy changes over time may provide insights into developmental periods during which interventions (pharmacological and behavioral) may be most beneficial. While this study revealed the value of diffusion imaging to understanding neuroanatomy in DS, an important methodological aspect to consider in future studies focused on morphometric measurements with DTI is to acquire diffusion data with acquisition strategies that allow for removal of spurious EPI distortions. The most promising approach to achieve this is to acquire data with reversed polarity of the phase encoding blips, and then utilize software tools to produce morphologically-faithful diffusion weighted images^64^.

In conclusion, the current study, which is the first to utilize DTI to describe both local white matter microstructure (FA and MD) and morphometry (DTBM) in DS, supports the utility of DTI, and DTBM in particular, for describing local differences in white matter volume, rather than attending only to possible differences in FA and MD. By utilizing DTBM, we were able to identify significant hypoplasia in fronto-pontine-cerebellar and olivo-cerebellar pathways in young people with DS, a novel finding in the DS neuroimaging literature. Future research on these pathways and relevant genes in humans with DS and murine models is warranted. Such research may provide important insights into the neurobiological underpinnings of the significant learning challenges faced by individuals with DS.

## Supplementary information

Supplemental Material.

## Data Availability

Data available upon request through protocol PI, Armin Raznahan, MD, Ph.D. Contact corresponding author with inquiries. Software used in this analysis is available for download: www.tortoisedti.org.
