# Supplementary material for: Hypoplasia of cerebellar afferent networks in Down syndrome revealed by DTI-driven tensor based morphometry
Source: Sci Rep. 2020 Mar 25;10:5447. doi: 10.1038/s41598-020-61799-1 (PMC7096514; doi:10.1038/s41598-020-61799-1)
Supplement: Supplementary file 1 — Supplemental Material. [file 41598_2020_61799_MOESM1_ESM.docx]

**Hypoplasia of cerebellar afferent networks in Down syndrome revealed by DTI-driven tensor based morphometry**

**SUPPLEMENTAL MATERIAL**

Nancy Raitano Lee^1,*, +^, Amritha Nayak^2,+,^ M. Okan Irfanoglu^2^, Neda Sadeghi^2^, Catherine J. Stoodley^3^, Elizabeth Adeyemi^4^, Liv S. Clasen^5^, and Carlo Pierpaoli^2^

^1^Drexel University, Department of Psychology, Philadelphia, PA, 19104, USA

^2^National Institute of Biomedical Imaging and Bioengineering, NIH, Quantitative Medical Imaging Section, Bethesda, MD, 20892, USA

^3^American University, Department of Psychology, Washington DC, 20016, USA

^4^Alabama College of Osteopathic Medicine, Dothan, AL, 36303, USA

National Institute of Mental Health, NIH, Developmental Neurogenomics Unit, Human Genetics Branch, Bethesda, MD, 20892, USA

^*^[nrl39@drexel.edu](mailto:nrl39@drexel.edu)

^+^these authors contributed equally to this work

METHOD

**Participants**

Participants with Down syndrome (DS):

Participants in the current investigation included 15 young people with DS. These participants were drawn from a sample of young people (n=54) who were enrolled in a larger research program focused on brain development in youth with DS ^1^. From this larger sample of 54 individuals, 6 participants dropped out of the research program prior to completing any MR imaging (due to family over-commitment or illness). Of the remaining 48 participants, 45 completed some scanning without sedation. The other 3 participants did not pass mock scanning procedures, and thus did not complete any study scanning procedures. Because DTI acquisition was completed at the end of a ~45-minute image acquisition sequence that included 12 minutes of clinical scans, a five-minute MPRAGE scan, and a five-minute resting state scan prior to DTI acquisition, only 20 participants with DS completed DTI scanning. These 20 participants were matched to 33 TD participants groupwise on chronological age, sex ratio, and family background characteristics (i.e., socioeconomic status as measured by the Hollingshead 2-factor index).

Prior to processing DTI data, the 20 acquired DTI scans for the DS group were inspected to ascertain quality utilizing procedures from the NIH Normal Brain Development Study^2^. Following this QC process, 15 youth with DS were found to have useable DTI scans. These individuals are the focus of the current investigation. A comparison of participants in the DS group with useable DTI data (n=15) and the participants with DS without useable DTI data (either due to quality control failure or inability to complete imaging procedures; n=33) revealed that groups differed significantly on age (DS group with useable DTI data (n=15): M = 17.02; SD= 5.47; DS group without useable DTI data (n=33): M = 12.18, SD = 5.16; t(46)=2.96; p<.005) but not sex (DS group with useable DTI data: 53% Male; DS group without useable DTI data: 58% Male; *p*>.7) or nonverbal IQ (DS group with useable DTI data: M = 59.53, SD = 15.19; DS group without useable DTI data: M = 52.67, SD = 17.65; *p*>.2)^[[1]](#footnote-1)^.

All participants with DS were diagnosed with Trisomy 21 via genetic testing per parent report. Of the 15 participants included in the current study, 12 cases were confirmed via direct testing as a part of this study. None of these participants were found to be mosaic. Of the remaining three cases (who elected not to complete genetic testing due to their child’s reluctance to give blood), two families provided copies of their child’s genetic testing results. One family was not able to locate these results but reported that their child was diagnosed with Trisomy 21. Exclusion criteria for the DS group included a history of acquired head injury or other condition that would cause gross brain abnormalities. However, one participant was included who had a well-controlled seizure disorder. See Lee et al. ^1^ for details.

Typically Developing (TD) Participants:

Following procedures outlined in Giedd *et al.*^3^, typically developing control participants were screened by phone prior to study enrollment to exclude for psychiatric or learning difficulties as well as acquired brain injury. A sample of 33 control participants, who were participating in the healthy volunteer program at the NIH, were selected and matched groupwise to DS participants on the following characteristics: age, proportion of males and females, and socioeconomic status using the Hollingshead 2-Factor Index. Of these 33 participants, 29 had usable DTI data following the QC procedure used to assess the scans for the DS participants. A comparison of participants with (n=29) and without (n=4) useable DTI data revealed no significant differences in age (Group with DTI data: M=16.13; SD= 6.64; Group without DTI data: M = 14.5, SD = 4.04; p>.4), sex (Group with DTI data: 49% Male; Group without DTI data: 75% Male; *p*>.2) or nonverbal IQ (Group with DTI data: M = 113.59, SD = 16.27; Group without DTI data: M = 117.00, SD = 14.49; *p*>.6).

Fifteen of the 29 TD participants were then matched carefully to the DS group on sex and age. These 15 participants comprised the TD control group for the current study. The other 14 TD participants’ scans were used to make the template for the current study. Demographic characteristics of the TD control and template samples are provided in Supplemental Table 1.

**Supplemental Table 1.** *Demographic characteristics of the group used to create the diffusion tensor (DT) template and the typically developing comparison group*

|  | **Template group**  (*n*=14) | | | | | | **TD control group** (*n*=15) | | | | | | |
| --- | --- | --- | --- | --- | --- | --- | --- | --- | --- | --- | --- | --- | --- |
|  |  |  |  |  | | | | |  | | |  | |
|  | *M* | *SD* | *Range* | | *M* | | | | | *SD* | | | *Range* |
|  |  |  |  | |  | | | | |  | | |  |
| Age | 14.44 | 6.44 | 5-24 | | 17.78 | | | | | 6.12 | | | 6-24 |
|  |  |  |  | |  | | | | |  | | |  |
| Nonverbal IQ | 115.57 | 14.15 | 85-140 | | 115.80 | | | | | 18.80 | | | 81-151 |
|  |  |  |  | |  | | | | |  | | |  |
| SES (Hollingshead)^1^ | 43.18 | 26.56 | 20-115 | | 33.38 | | | | | 13.43 | | | 20-51 |
|  |  |  |  | | |  | | |  | | |  | |
|  |  |  |  |  | | | | |  | | |  | |
|  |  |  |  |  | | | | |  | | |  | |
|  | *N* | *%* |  |  | | | | *N* | | | *%* | | |
| Sex: Female | 9 | 64.29 |  |  | | | | 7 | | | 46.67 | | |
|  |  |  |  |  | | | |  | | |  | | |
|  |  |  |  |  | | | |  | | | |  | |

Note: Groups did not differ on age, nonverbal IQ, SES, or sex ratio; ^1^SES data are missing for 3 participants in the template group and 2 participants in the TD group.


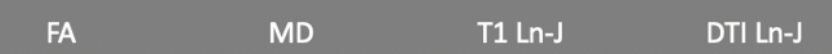

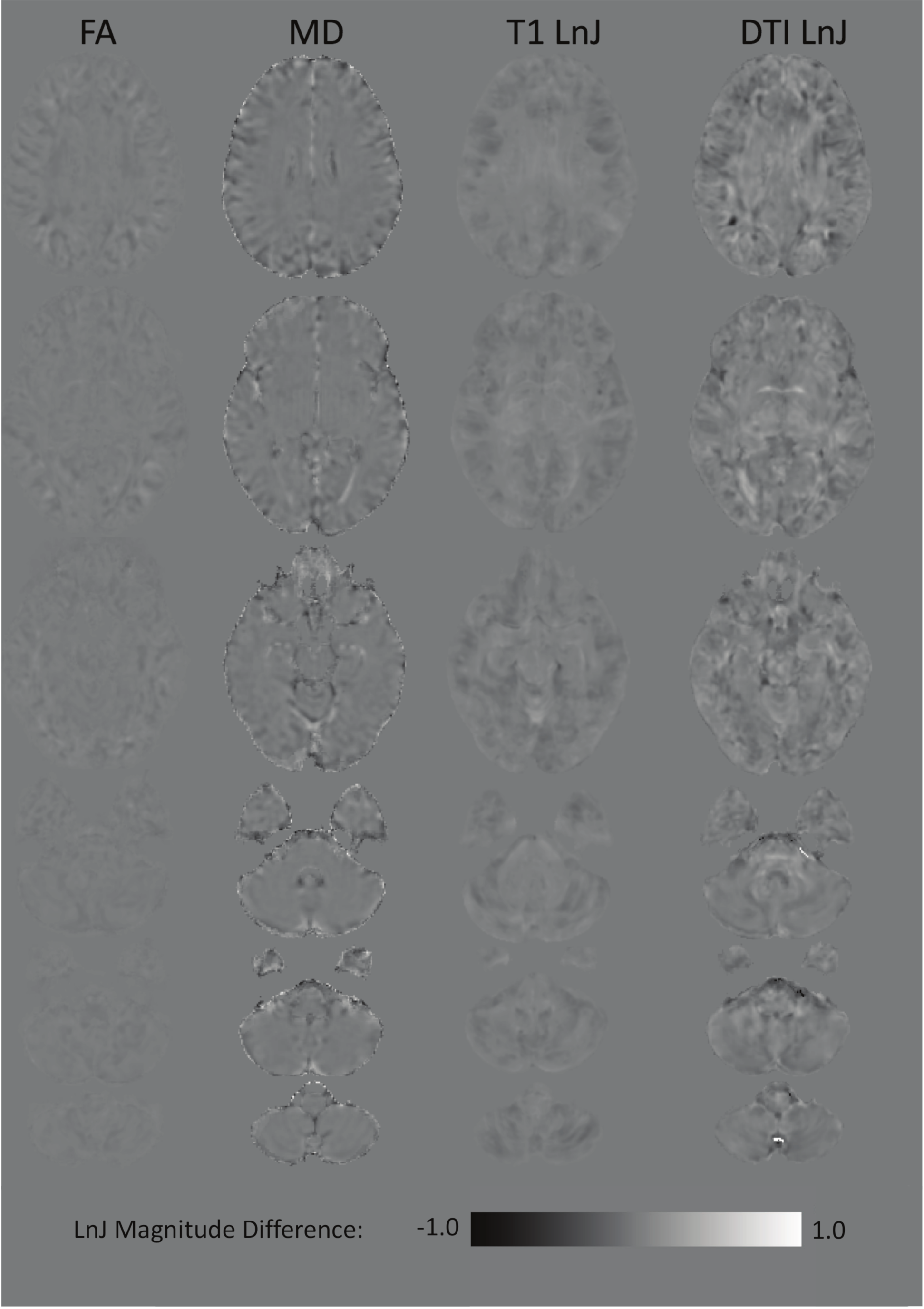


**Supplemental Figure 1.** *Magnitude difference for TD control group – TD Template group for FA, MD, T1Ln-J, and DTI Ln-J.* The gray level in the background correspond to zero, gray levels darker than the background indicate negative values and gray levels brighter than background indicate positive values. All values are scaled from -1 (black) to +1 (white). Ln-J and FA values are dimensionless, MD values are in units of 10^-3^ mm^2^/s

**Results: Supplemental Material**


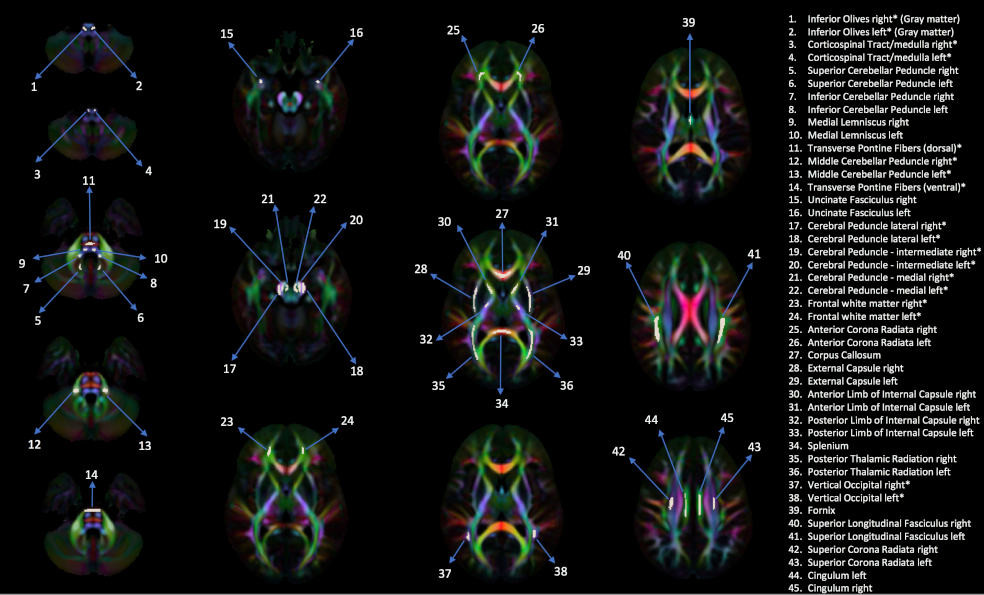


**Supplemental Figure 2.** *White matter structures defined directly on the DEC maps of the DT template.* 15 regions (5-10,15,16, 25-36,39-45) were defined based on the regions described under the JHU white matter atlas (Oishi et al. 2008) and 10 regions (*1-4, *11-14, *17-24, *37,*38) were defined that were relevant for this analysis but that are not defined on the JHU atlas. Regions were defined on left (l) and right (r) hemispheres and the values from l,r were combined for the values reported.

­­

References for Supplemental Material

1. Lee NR, Adeyemi EI, Lin A, et al. Dissociations in Cortical Morphometry in Youth with Down Syndrome: Evidence for Reduced Surface Area but Increased Thickness. *Cereb Cortex.* 2016;26(7):2982-2990.

2. Walker L, Chang LC, Nayak A, et al. The diffusion tensor imaging (DTI) component of the NIH MRI study of normal brain development (PedsDTI). *Neuroimage.* 2016;124(Pt B):1125-1130.

3. Giedd JN, Lalonde FM, Celano MJ, et al. Anatomical brain magnetic resonance imaging of typically developing children and adolescents. *J Am Acad Child Adolesc Psychiatry.* 2009;48(5):465-470.

1. Note that in the group without usable DTI data, IQ data were available for 29 of the 33 participants [↑](#footnote-ref-1)
